# Transcriptome instability as a molecular pan-cancer characteristic of carcinomas

**DOI:** 10.1186/1471-2164-15-672

**Published:** 2014-08-10

**Authors:** Anita Sveen, Bjarne Johannessen, Manuel R Teixeira, Ragnhild A Lothe, Rolf I Skotheim

**Affiliations:** Department of Cancer Prevention, Institute for Cancer Research, The Norwegian Radium Hospital, Oslo University Hospital, P.O. Box 4953, Nydalen, Oslo NO-0424 Norway; Centre for Cancer Biomedicine, Faculty of Medicine, University of Oslo, P.O. Box 1078, Blindern, Oslo NO-0316 Norway; Department of Genetics, Portuguese Oncology Institute, Rua Dr. António Bernardino de Almeida, Porto, 4200-072 Portugal; Cancer Genetics Group, Research Centre of the Portuguese Oncology Institute, Rua Dr. António Bernardino de Almeida, Porto, 4200-072 Portugal; Institute of Biomedical Sciences Abel Salazar, University of Porto, Rua de Jorge Viterbo Ferreira n.° 228, Porto, 4050-313 Portugal

**Keywords:** Alternative splicing, Carcinomas, Exon microarray, Splicing factor, Tissue specificity

## Abstract

**Background:**

We have previously proposed transcriptome instability as a genome-wide, pre-mRNA splicing-related characteristic of colorectal cancer. Here, we explore the hypothesis of transcriptome instability being a general characteristic of cancer.

**Results:**

Exon-level microarray expression data from ten cancer datasets were analyzed, including breast cancer, cervical cancer, colorectal cancer, gastric cancer, lung cancer, neuroblastoma, and prostate cancer (555 samples), as well as paired normal tissue samples from the colon, lung, prostate, and stomach (93 samples). Based on alternative splicing scores across the genomes, we calculated sample-wise relative amounts of aberrant exon skipping and inclusion. Strong and non-random (*P* < 0.001) correlations between these estimates and the expression levels of splicing factor genes (n = 280) were found in most cancer types analyzed (breast-, cervical-, colorectal-, lung- and prostate cancer). This suggests a biological explanation for the splicing variation. Surprisingly, these associations prevailed in pan-cancer analyses. This is in contrast to the tissue and cancer specific patterns observed in comparisons across healthy tissue samples from the colon, lung, prostate, and stomach, and between paired cancer-normal samples from the same four tissue types.

**Conclusion:**

Based on exon-level expression profiling and computational analyses of alternative splicing, we propose transcriptome instability as a molecular pan-cancer characteristic. The affected cancers show strong and non-random associations between low expression levels of splicing factor genes, and high amounts of aberrant exon skipping and inclusion, and *vice versa*, on a genome-wide scale.

**Electronic supplementary material:**

The online version of this article (doi:10.1186/1471-2164-15-672) contains supplementary material, which is available to authorized users.

## Background

The four major types of cancer, lung cancer, breast cancer, colorectal cancer, and prostate cancer, constitute approximately 40% of cancer cases world-wide, with more than 5 million new diagnoses and 2.7 million related deaths every year [1]. These are all epithelial cancers and are commonly characterized by genomic instability [2]. Genomic instability is described as an enabling hallmark of cancer, generating random mutations which may also hit cancer critical genes [3]. At the nucleotide level, genomic instability can occur as frequent mutations of short nucleotide repeats dispersed throughout the genome, referred to as microsatellite instability, and results from a defective mismatch repair system [4–6]. Microsatellite instability has been described in several solid cancer types, including cancers of the colon, endometrium, stomach, and lung [7–11], and is associated with good prognosis in colorectal cancer [9, 12, 13]. Chromosomal instability, characterized by numerical and structural chromosome changes, is common in solid cancers, but the causes for this molecular phenotype remain mostly unknown. Measured as aneuploidy, chromosomal instability has been found to be associated with poor prognosis in all four major types of carcinomas [14–17]. In addition to genomic instability, epigenome abnormality has also been described in several cancer types, although most prominent in colorectal cancer [18]. Cancers with the CpG island methylator phenotype have frequent DNA hypermethylation in gene promoter regions, often resulting in gene silencing [19]. Recently, we described genome-wide instability acting also on the level of the transcriptome in colorectal cancer, affecting the pre-mRNA splicing process [20]. This transcriptome instability (TIN) was characterized by large variation in amounts of aberrant inclusion and skipping of exons in colorectal cancer samples. These amounts were shown to be strongly associated with both the expression levels of pre-mRNA splicing factors and poor prognosis for patients with colorectal cancer [20].

RNA splicing is a tightly regulated and highly tissue specific process that can occur by a number of different modes [21]. Alternative splicing modes include differential inclusion of whole exons (cassette exons) or parts of exons (alternative 5’ or 3’ splice sites), intron retention, mutual exclusion of cassette exons, alternative ordering of exons (exon scrambling) [22], and splicing of exons encoded by different genes (*trans*-splicing) [23]. Such alternative splicing is a major source of functional diversity in the human genome [24]. Nearly all multi-exon genes are alternatively spliced, predicted to undergo on average seven alternative splicing events during tissue development and across tissue types [25, 26]. In cancer, the RNA splicing pathway is commonly disrupted, as evident by aberrant, disease-specific splicing patterns [27]. There is a large collection of proteins regulating the splicing process, and genes encoding these splicing factors have been identified as cancer critical genes, as is the case with *SRSF1* [28]. Splicing factors have been found to be both differentially expressed [29] and commonly mutated in cancer [30–32]. Hence, there is tremendous potential for splicing variation in the cancer transcriptome.

In the present study, we describe TIN in several major types of malignancies, including breast cancer, cervical cancer, colorectal cancer, lung cancer, and prostate cancer. By analyzing exon microarray profiles for alternative splicing, we characterized the individual samples within the datasets for relative amounts of aberrant exon skipping and inclusion. In cancer types with TIN, we found strong and non-random associations between these amounts and the expression levels of splicing factors. Surprisingly, this association was intact in pan-cancer analysis of the TIN-cancers, indicating that the pronounced tissue specificity found in corresponding analyses across normal tissue types and paired cancer-normal samples was lost.

## Results

### Strong correlations between TIN-estimates and expression levels of splicing factors

Exon microarray profiles for 555 samples from ten different cancer datasets and 93 samples from four normal tissue datasets (Table 1) were analyzed for genome-wide alternative splicing variation. The lower and upper 1^st^ percentiles of alternative splicing (FIRMA) scores within each dataset were used as threshold values to designate exons as aberrantly spliced (range −2.4 to −2.0 and 1.8 to 2.1, respectively; log2-scale; Table S1 in Additional file 1). Sample-wise amounts of aberrant exon skipping and inclusion were calculated as the number of exons exceeding these threshold values (Additional file 1: Table S1). These sample-wise amounts of aberrant splicing, relative to the average amount within the dataset, are referred to as TIN-estimates. The TIN-estimates and range of TIN-estimates (the difference between the sample with the highest and lowest estimate) were similar within all datasets (average range 3.4; Additional file 1: Table S1). There was no correlation in TIN-estimates between paired cancer and normal samples from the colon (n = 19), lung (n = 20), prostate (n = 29), or stomach (n = 23; Additional file 1: Figure S1). In the breast cancer dataset, there were significantly lower TIN-estimates in estrogen and progesterone receptor positive than negative samples (*P* = 0.04, independent samples t-test; Additional file 1: Figure S2). Also, there were significant differences in TIN-estimates between histological subtypes of both cervical cancer and lung cancer series II. In the cervical cancer dataset, adenocarcinomas had higher TIN-estimates than squamous cell carcinomas (*P* = 0.002), whereas the opposite was found in lung cancer series II (*P* < 0.001; Additional file 1: Figure S2). There were no associations between TIN-estimates and sample characteristics in any of the other datasets (characteristics listed in Table 1).

**Table 1:** **Samples included in the study**

Tissue	Samples	Histology	Other sample characteristics	GEO accession number	Literature references
Breast cancer	84	-	Hormone receptor status: HER2-positive (n = 35); ER/PR-positive (n = 25); ER/PR/HER2-negative (n = 24)	GSE16534	[45]
Cervical cancer	28	Squamous cell carcinoma (n = 19); adenocarcinoma (n = 9)	-	GSE27388	[44]
Colorectal cancer series I	26	Adenocarcinoma	-	GSE16534	[45]
Colorectal cancer series II	101^a^	Adenocarcinoma	Stage: Stage I (n = 28); stage II (n = 34); stage III (n = 26); stage IV (n = 13)	GSE24550 (n = 55); GSE29638 (n = 46)	[20, 49]
			MSI-status: MSI-high (n = 21); MSS/MSI-low (n = 77); NA (n = 3)		
Gastric cancer	25	Adenocarcinoma	Stage: Stage I (n = 8); stage II (n = 4); stage III (n = 5); stage IV (n = 8)	GSE13195	-
Lung cancer series I	20	Non-small-cell lung adenocarcinoma	Stage: Stage I (n = 12); stage II (n = 3); stage III (n = 5)	GSE12236	[48]
Lung cancer series II	43	Non-small-cell lung adenocarcinoma (n = 21) and squamous cell carcinoma (n = 22)	-	GSE16534	[45]
Neuroblastoma	47	-	Stage: stage I (n = 10); stage IV (n = 37)	GSE27608	[43]
Prostate cancer series I	131	Adenocarcinoma Gleason grade: Grade 5 (n = 1); grade 6 (n = 77); grade 7 (n = 42), grade 8 (n = 7); grade 9 (n = 4)	Pathologic T stage: stage 2 (n = 85); stage 3 (n = 40); stage 4 (n = 6)	GSE21034	[47]
Prostate cancer series II	50^a^	Gleason grade: Grade 5 (n = 2); grade 6 (n = 15); grade 7 (n = 32); grade 9 (n = 1)	Pathologic T stage: stage 2 (n = 26), stage 3 (n = 24)	GSE42954	[46]
Normal colonic mucosa	21^a^		19 samples corresponding to tumors from GSE24550 and GSE29638	GSE42690 (n = 19); GSE29638 (n = 2)	[20, 49]
Normal lung	20		Corresponding to tumors from GSE12236	GSE12236	[48]
Normal prostate	29		Corresponding to tumors from GSE21034	GSE21034	[47]
Normal stomach	23^b^		Corresponding to tumors from GSE13195	GSE13195	-

^a^Patient samples analyzed in-house ^b^Two samples from the GEO entry (GSM333256 and GSM333270) were excluded due to failure of reading the raw data files. *Abbreviations: GEO* Gene Expression Omnibus, *MSI* microsatellite instability, *MSS* microsatellite stable, *NA* not available *T* tumor.

**Figure 1:**
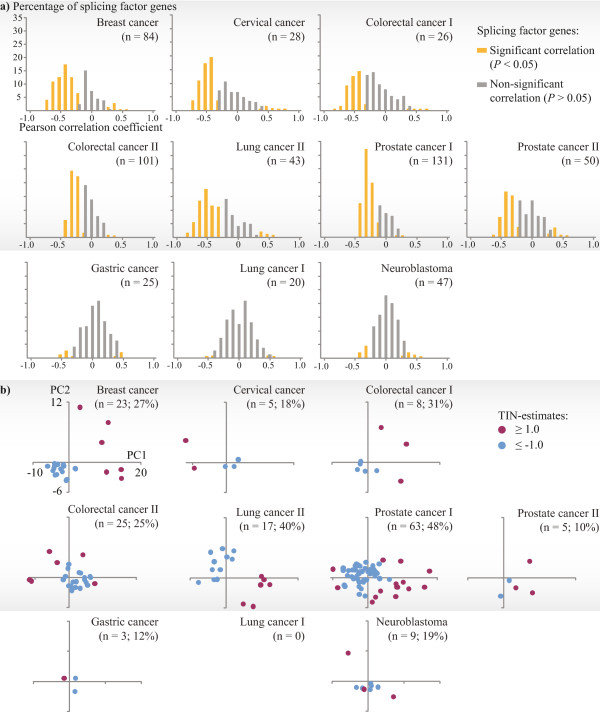
**TIN**-**estimates are associated with the expression levels of splicing factor genes in seven cancer datasets.** **(a)** In seven of the cancer datasets (plots with grey background) there were strong and significant correlations (horizontal axes) between TIN-estimates and the expression levels of ≥41% of the totally 280 splicing factor genes. In the other three datasets, the correlations were mainly non-significant. Yellow and grey bars represent the percentages of splicing factor genes with significant and non-significant correlations, respectively. **(b)** In two-dimensional principal components analysis based on the expression levels of splicing factor genes (n = 280), samples were generally separated according to TIN-estimates (only samples with TIN-estimates ≥ ±1.0 were included, the number and percentages of samples are indicated for each dataset) in the same seven datasets (with grey background). In colorectal cancer series II and prostate cancer series I, the separation was not complete. In the gastric cancer dataset and lung cancer series I, there were few samples with TIN-estimates ≥ ±1.0. In the neuroblastoma dataset, there was no clear separation. PC, principal component.

For samples in seven of the cancer datasets (breast cancer, cervical cancer, colorectal cancer series I and II, lung cancer series II, and prostate cancer series I and II), the sample-wise TIN-estimates correlated strongly with the expression levels of pre-mRNA splicing factors (n = 280). The amount of significantly correlated splicing factor genes (Pearson correlation, *P* < 0.05) ranged from 41% (colorectal cancer series I) to 70% (prostate cancer series I) within the datasets (Figure 1a). There was a clear preference for negative correlation, and the average correlation coefficients for the significantly correlated genes ranged from −0.5 to −0.3 (colorectal cancer series I and II, respectively). In these datasets, samples were also separated according to TIN-estimates when performing unsupervised hierarchical clustering analyses by the expression levels of all splicing factors (Additional file 1: Figure S3). Principal components analyses based on splicing factor gene expression corroborated these results (Figure 1b). In general, samples with low TIN-estimates (≤ − 1.0) were separated from samples with high TIN-estimates (≥1.0), although not perfectly in colorectal cancer series II and prostate cancer series I. This association between TIN-estimates and expression levels of splicing factors was less clear in the other three cancer datasets (gastric cancer, lung cancer series I, and neuroblastoma; Figure 1). Here, the amount of significantly correlated splicing factor genes ranged from 2% (lung cancer series I) to 10% (neuroblastoma). Analyses of the datasets with normal samples indicated associations between TIN-estimates and expression levels of splicing factors also in non-cancerous tissues, with strongest associations in normal colonic mucosa (Additional file 1: Figure S4). This variance between the datasets showed no associations to quality control metrics of the exon microarray data (Additional file 1: Figures S5 and Additional file 1: Figure S6). The three cancer datasets not showing associations between TIN-estimates and expression levels of splicing factors had similar quality control metrics to the other datasets, whereas the dataset showing most deviance during quality control (cervical cancer), showed strong associations.

**Figure 2:**
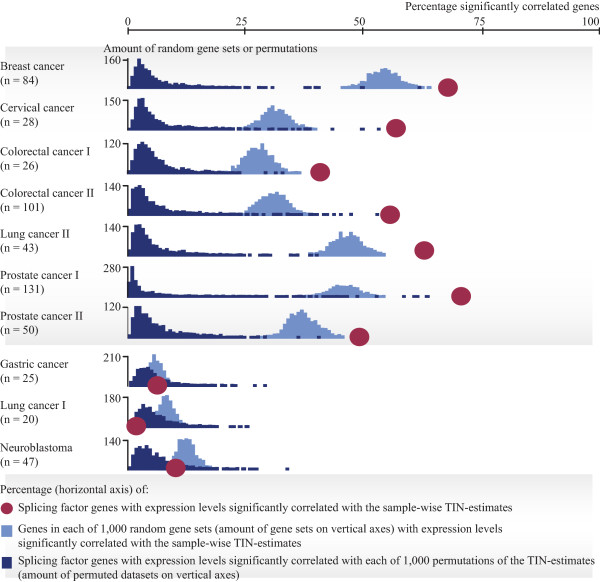
**Correlation between TIN** **-** **estimates and splicing factor expression compared with random gene sets and permuted TIN-** **estimates.** In seven of the cancer datasets (with grey background), high percentages of the 280 splicing factor genes had expression levels that were significantly correlated (*P* < 0.05; Pearson correlation) with the sample-wise TIN-estimates (41% to 70%; red dots; horizontal axis). This was more than expected by chance, as compared with genes in each of 1,000 random sets of 280 genes (bar graphs in light blue) and with 1,000 permutations of the TIN-estimates (bar graphs in dark blue). In the other three cancer datasets (no background), the percentages of significantly correlated splicing factor genes were much lower and not higher than expected by chance.

To test whether the observed associations between TIN-estimates and expression levels of splicing factors were greater than expected by chance, comparisons were made with random gene sets of equal size (n = 1,000 sets of 280 genes; calculation of percentage of genes in each random gene set with expression levels significantly correlated with the TIN-estimates), and permutations of the TIN-estimates in each dataset (n = 1,000 permutations; calculation of percentage of splicing factor genes with expression levels significantly correlated with each round of permutation). Corresponding with the results above, the same seven cancer datasets that showed strongest associations, also had higher amounts of splicing factor genes with expression levels significantly correlated with the TIN-estimates than was expected by chance (*P* < 0.001), as opposed to the other three cancer datasets (Figure 2). The same results were obtained also when analyzing correlation strengths (Additional file 1: Figure S7). Interestingly, all the normal tissue datasets also had higher percentages of splicing factor genes with expression levels that were significantly correlated with the TIN-estimates than expected by chance (Additional file 1: Figure S8). The association was particularly strong in normal colonic mucosa, where 56% of the splicing factors were significantly correlated.

Also in comparison with all gene sets included in the Molecular Signatures Database v3.1 (MSigDB; annotated with Gene Ontology terms; n = 1,454 gene sets), as well as with the full genome (n = 17,881 genes), the splicing factor gene set (and other splicing-related gene sets included in MSigDB) had high amounts of genes with expression levels significantly correlated with TIN-estimates in the same seven cancer datasets (Additional file 1: Figure S9).

The percentage of TIN-samples (samples with TIN-estimates ≥ ±1.0) in each dataset varied from 0 in lung cancer series I to 48% in prostate cancer series I (Figure 1b). The datasets with the highest percentages also had higher amounts of splicing factor genes with expression levels significantly correlated with the TIN-estimates (Additional file 1: Figure S10). We consider this to strengthen the notion that tissue and cancer types with high variation in splicing also have a correspondingly high variation in splicing factor expression.

### Inverse relationship between TIN-estimates and expression levels of splicing factors

**Figure 3:**
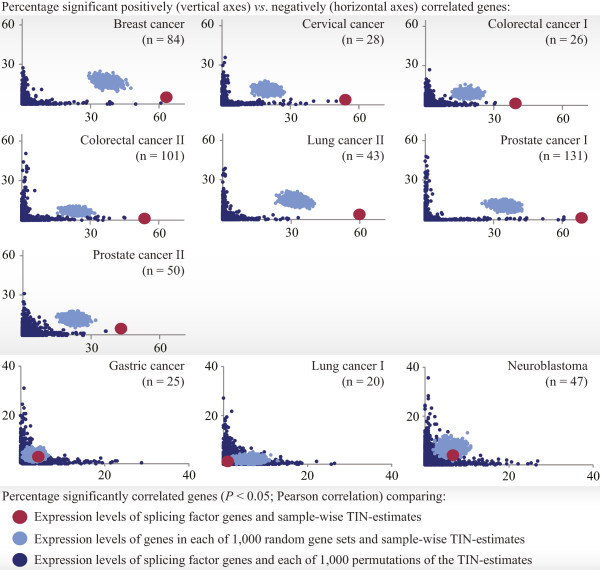
**Negative correlation between TIN** **-** **estimates and splicing factor expression in the cancer datasets.** In the seven cancer datasets (grey background) with strong associations between TIN-estimates and expression levels of splicing factors (n = 280), the relationship was inverse, with a much higher percentage of significant negatively (horizontal axes) than positively (vertical axes) correlated splicing factor genes (red). This shift was higher than expected by chance, as demonstrated by comparisons with genes in each of 1,000 random sets of 280 genes (light blue), and with each of 1,000 permutations of the TIN-estimates (dark blue). This significant shift towards negative correlation was not found in the three cancer datasets with weak associations between TIN-estimates and splicing factor expression (no background).

In the seven cancer datasets with strong associations between TIN-estimates and expression levels of splicing factors, the associations were mainly inverse. The amount of genes with expression levels that were negatively correlated with the TIN-estimates (≥89% of the significantly correlated splicing factor genes in all seven datasets) was much larger than the amount of positively correlated genes, with ratios ranging from 8 in prostate cancer series II to 97 in prostate cancer series I. This ratio of negative *vs*. positive correlations was significantly higher than expected by chance (again compared with random gene sets and permutations of the amounts of aberrant exon usage; *P* < 0.001; Figure 3). This significant shift towards negative correlation was not found in the three cancer datasets with weak associations between TIN-estimates and expression levels of splicing factors (ratios ranging from 1 to 2; Figure 3). In the normal tissue types, there was also a significant, but less pronounced shift towards negative correlation. Here, the ratios of the amounts of significant negatively *vs*. positively correlated splicing factor genes ranged from 4 (normal colonic mucosa) to 30 (normal stomach; Additional file 1: Figure S11).

Also in comparison with gene sets in MSigDB, as well as with the full genome, the splicing factor gene set (and other splicing-related gene sets included in MSigDB) had a strong shift towards negative correlation in the same seven cancer datasets (Additional file 1: Figure S12).

### Transcriptome instability as a pan-cancer characteristic

**Figure 4:**
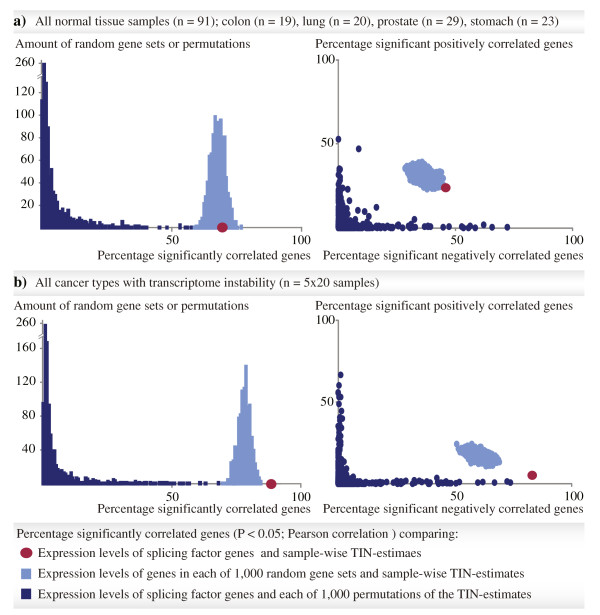
**Correlation between TIN** **-** **estimates and splicing factor expression in pooled tissue types.** The left and right plots correspond to plots in Figures 2 and 3, respectively. **(a)** In pooled analyses of samples from four different normal tissues, the percentage of splicing factor genes (totally 280 genes; red dots) with expression levels that were significantly correlated with the TIN-estimates (*P* < 0.05; Pearson correlation), was not higher than expected by chance, as demonstrated by comparison with genes in each of 1,000 random sets of 280 genes (light blue) and with 1,000 permutations of the TIN-estimates (dark blue; left plot). The ratio between the amounts of significant negatively and positively correlated splicing factor genes was 2 (right plot). **(b)** Contrarily, across 20 randomly selected samples from each of five different cancer types with strong associations between TIN-estimates and splicing factor expression (breast cancer, cervical cancer, colorectal cancer series II, lung cancer series II, and prostate cancer I) the percentage of splicing factor genes with expression levels that were significantly correlated was higher than expected by chance (left plot). Also, there was a significant shift towards negative correlation (the ratio between the amounts of splicing factors genes with expression levels that were significant negatively and positively correlated was 17; right plot).

Although the individual normal tissue datasets showed strong correlations between the TIN-estimates and expression levels of splicing factors, this association was lost in pooled analyses of the normal tissues (n = 91) of four different origins. Again, this was tested by calculating the correlation between TIN-estimates and expression levels of splicing factors across all the different normal tissue types, and comparing with random gene sets and permutations of the TIN-estimates (Figure 4a). This is compliant with the previously described tissue specificity in both splicing patterns [25, 33] and expression levels of splicing factors reviewed in [34]. The same results were found from analysis of the paired cancer-normal sample sets (except for in the colon; Additional file 1: Figure S13), again in agreement with the fact that pre-mRNA splicing is differentially regulated also between cancer and normal tissues [27]. Surprisingly, when doing the same comparisons across the cancer types with strong associations between TIN-estimates and splicing factor expression, the strong and non-random associations prevailed (*P* < 0.001; Figure 4b; for this analysis, 20 samples were randomly selected from each of the five cancer types, to assess splicing patterns across similar numbers of samples as for the normal tissues. For cancer types represented by two datasets, samples were selected from the datasets with paired normal samples, *i.e*. colorectal cancer series II and prostate cancer series I). In these pan-cancer analyses, there was a much higher amount of significant negatively than positively correlated splicing factor genes (232 genes compared with 14 genes, ratio 17; also this higher than expected by chance, compared with random gene sets and permutations of the TIN-estimates, *P* < 0.001; Figure 4b). When limiting the analyses to cancer samples for which paired normal samples were available, the results were corresponding. Non-random associations between TIN-estimates and splicing factor expression were found only in analyses across colorectal cancer series II and prostate cancer series I (Additional file 1: Figure S13). This indicates that TIN is a shared molecular characteristic across the affected cancer types.

## Discussion

We have previously proposed genome-wide instability of the transcriptome as a molecular, pre-mRNA splicing related characteristic of colorectal cancer. Here, we show data indicating that the transcriptomes of several, but not all, other types of solid tumors also have this feature. Common cancers of the breast, cervix, large bowel, lung, and prostate all show large variation in sample-wise amounts of aberrant exon skipping and inclusion (TIN-estimates) that are significant negatively correlated with the expression levels of splicing factor genes. Such associations were also found within healthy tissue types, although to less extent. This is consistent with the fact that most tissues exhibit great and tightly regulated splicing variation. However, the splicing patterns were clearly distinct between healthy tissues and their malignant counterparts, as shown by discordant TIN-estimates between paired cancer and normal samples in all the four different tissue types analyzed. This is in compliance also with the original TIN-report, showing that colorectal cancers have higher TIN-estimates than normal colonic mucosa [20]. Furthermore, in analysis across the various healthy tissues, the correlation between TIN-estimates and expression levels of splicing factors was not greater than expected by chance. This indicates a failure to detect a common pattern of regulation, and that the splicing process is predominantly tissue specific in healthy tissues. Interestingly, in pan-cancer analyses, the strong associations with expression levels of splicing factors prevailed. These results suggest that TIN is a pan-cancer characteristic, clearly distinguished from normal splicing variation and splicing factor expression.

The importance of molecular pan-cancer analyses has recently been illuminated by The Cancer Genome Atlas Pan-Cancer project [35]. The recognition that cancers from separate organs may have shared molecular features, while cancers from the same organ may be distinct, has great potential influence both biologically, on our understanding of the tumorigenic process, and clinically, for example for more personalized and targeted treatment strategies. Furthermore, pan-cancer analyses may aid in the identification of novel cancer-critical genes not disclosed in individual cancer types because of low mutation rates [36]. With regard to pan-cancer analysis of alternative splicing, mutation of the splicing factor gene *U2AF1* has been shown to result in both distinct and equal aberrant splicing events in lung adenocarcinoma and acute myeloid leukemia [37], indicating both specific and common regulation in the two cancer types. Here, from integrated expression analyses of a comprehensive collection of splicing factor genes, we have described TIN both within and across individual cancer types, suggesting that TIN is a general characteristic of cancer. Closer inspection of individual splicing factor genes revealed differences between the cancer types with respect to which genes were most closely associated with the aberrant splicing amounts (data not shown). No single splicing factor gene seceded in these pan-cancer analyses, suggesting that there is no dependency for the involvement of specific splicing factors in the establishment of TIN.

In this study, genome-wide analyses of alternative splicing were performed by computational analyses of exon-level microarray data. Although experimental work is needed to elucidate the functional mechanisms, an underlying biological explanation for the observed variation is suggested by the strong and non-random associations with the expression levels of splicing factors in several cancer types. In fact, this association increased with increasing splicing variation, as indicated by the correlation between the amount of TIN-samples per dataset (samples with TIN-estimates ≥ ±1.0) and the number of splicing factor genes with expression levels that were significantly correlated with the TIN-estimates. Also worth noting is the striking inverse nature, with the great majority of splicing factors having expression levels that were negatively correlated with the TIN-estimates. Altogether, this suggests a mechanism in which decreased expression levels of splicing factors result in more aberrant exon skipping and inclusion. The fact that the majority of splicing factors are involved in several cancer types suggests a genome-wide mechanism. This is consistent with previous reports showing a coordinated regulation of RNA splicing by numerous splicing factors [38].

To describe TIN as a genuine molecular pan-cancer characteristic, identification of individual, aberrant splicing events and functional validation of their correlations with splicing factor expression levels are warranted. Such analyses are complicated by the genome-wide nature of the described associations. No single splicing factor is likely to account for all the observed variation. This situation resembles the obstacles faced also when characterizing the now well established genetic and epigenetic genome-wide phenotypes (with the exception of microsatellite instability found in subgroups of certain cancer types). However, we anticipate that ongoing RNA sequencing efforts will be highly valuable in gaining further insights into the proposed TIN characteristic.

There are various non-biological factors that may have influenced the splicing variation observed in this study. Firstly, the analyses are sensitive to the sizes of the datasets, and the variation in TIN-estimates increased with sample numbers (data not shown). This is an inherent consequence of the analyses, as differential exon skipping and inclusion were detected relative to all included samples. Secondly, technical variation in the microarray data may also have contributed. Accordingly, thorough quality control of the data was performed, and the extent of correlations between splicing variation and expression levels of splicing factors in each dataset, was independent of various quality control metrics. However, the failure to detect the strong association between splicing variation and splicing factor expression in three of the cancer datasets (gastric cancer, lung cancer series I, and neuroblastoma) may be attributable to non-biological features of the data rather than inherent characteristics of the cancer types. In particular, the two datasets from lung cancer showed conflicting results, with strong associations between the sample-wise TIN-estimates and splicing factor expression in series II, but not in series I. Both these patient series consist of non-small-cell carcinomas, but series I comprises adenocarcinomas only, whereas series II also includes squamous cell carcinomas. Although the TIN-estimates were significantly higher in the squamous cell carcinomas than the adenocarcinomas in lung cancer series II, the subtypes individually showed strong associations between the TIN-estimates and the expression levels of splicing factors (data not shown). For the other two cancer types represented by two datasets, colorectal cancer and prostate cancer, strong associations were clearly indicated in both datasets.

Splicing factor encoding genes have previously been nominated as cancer-critical based on their altered expression levels [28]. Recently, individual splicing factor genes have also been found to be commonly mutated in different cancer types, and this has been shown to have important implications for carcinogenesis [30–32, 39, 40]. In this study, mutation analyses have not been conducted. Furthermore, only the splicing events aberrant skipping and inclusion of cassette exons, as well as intron retention, have been considered. Although these are the most common modes of splicing [41], a complete view of the genome-wide effects of splicing factor expression on the splicing process should also consider the other events regulated by splicing factors (alternative 5’ and 3’ splice sites, patterns of mutual exclusion, *trans*-splicing, and exon scrambling).

Although premature, the implications of TIN as a common and novel, genome-wide characteristic of epithelial cancers are intriguing and warrant further investigation. Similarly to the genetic and epigenetic genome-wide phenotypes that have been proven to be important clinical characteristics, allowing for sub-grouping of patients according to prognosis [13, 16] and tumor histology [42], TIN may also have clinical potential. Previously, we have shown that TIN is associated with adverse outcome for patients with stage II and III colorectal cancer [20].

## Conclusions

By computational analysis of alternative splicing based on exon-level microarray data, we show that common types of solid cancers (including breast, cervical, colorectal, lung, and prostate cancers) exhibit large variation among samples in amounts of aberrant skipping and inclusion that are non-randomly associated with the expression levels of splicing factors. Importantly, this type of transcriptome instability is a pan-cancer characteristic, as opposed to the tissue specificity prevailing in healthy tissues. Functional evaluation of the associations between this splicing pattern and the expression levels of splicing factors is needed to determine if transcriptome instability is a genuine phenotype of solid cancers.

## Methods

### Patient material

In this study, genome-wide expression data at the exon level for a comprehensive collection of 555 tissue samples from seven types of solid tumors, including the four most common, breast, colorectal, lung, and prostate, have been analyzed. Additionally, the study comprises expression data for a total of 93 normal tissue samples, including paired samples from cancers of the colon, lung, prostate, and stomach. All exon-level microarray datasets (Affymetrix HuEx-1_0-st-v2 arrays) with more than 20 cancer samples that have been made publically available by us and others have been included [20, 43–49], except 83 colorectal cancer samples in GSE24549, which we previously used to describe TIN [20]. An overview of the datasets, with available clinical information of the tissue samples, is found in Table 1. From each of colorectal cancer, lung cancer and prostate cancer, two datasets have been included and are referred to as series I and II, respectively. Colorectal cancer series II is an in-house, consecutive series of stage I – IV cancers (90% inclusion rate), collected at Aker University Hospital, Oslo, Norway, between 2005 and 2007. This series also comprises 21 normal colonic mucosa samples (including 6 that have not been published before) taken from disease-free areas distant to the primary tumors (19 matched sample pairs are included). Prostate cancer series II is also an in-house series, consisting of 50 primary tumor samples from a consecutive series of 200 clinically localized cancers treated with radical prostatectomy at the Portuguese Oncology Institute, Porto, Portugal.

The analysis of the additional samples published herein has been approved by the institutional review boards (the Regional Committee for Medical and Health Research Ethics, number 1.2005.1629; and reference [46]), which involves that informed consent is obtained from patients being enrolled to the study.

### Alternative splicing analysis

All 648 samples have been analyzed for gene expression at the exon level by the Affymetrix GeneChip Human Exon 1.0 ST Array (Affymetrix Inc, Santa Clara, CA, USA). This array contains approximately 6 million different probes, providing genome-scale, exon-resolution expression measures. Each probe set consists of an average of four probes and generally corresponds to one exon. For each sample, 284,258 probe sets belonging to the ‘core’ set of probes targeting well annotated exons were analyzed, using an annotation file custom-made for alternative splicing analysis with the Finding Isoforms using Robust Multichip Analysis (FIRMA) algorithm (HuEx-1_0-st-v2,coreR3,A20071112,EP.cdf) [50, 51].

Probe cell intensity (CEL) files storing raw data for each of the samples were used as input for preprocessing and alternative splicing analysis with the FIRMA algorithm implemented in the R software environment [52] (GEO accession numbers of both public and previously unpublished data are indicated in Table 1). This provided the basis for calculations of sample-wise amounts of aberrant exon skipping and inclusion within the datasets, as we have also previously described [20]. The first two steps of FIRMA follow the RMA approach [53] for background correction of individual probes based on GC-content, and for inter-chip quantile normalization. Then, alternative splicing scores (FIRMA scores) are calculated for each individual exon in each individual sample, as the deviance between the exon level and corresponding overall gene level expression measures. These scores represent the residuals from fitting gene level models to the exon level data, according to the mapping provided in the custom-made annotation file. The FIRMA scores were log2-transformed. Strong positive and negative scores reflect differential exon inclusion and skipping, respectively, compared with the rest of the samples in the dataset. The lower and upper 1^st^ percentiles of FIRMA scores across each dataset (Additional file 1: Table S1) were used as thresholds for identification of deviating (aberrant) exon skipping and inclusion, respectively. Sample-wise amounts of exons with aberrant splicing patterns were summarized from the exons exceeding these threshold values. Amounts of aberrant exon skipping or inclusion per sample are presented on a log2-scale, relative to the average sample-wise amount within the individual datasets. As a measure of TIN, aberrant exon skipping and inclusion were summarized. This TIN-estimate thus represents the total amounts of aberrant exon inclusion and skipping per sample (on a log2-scale), relative to the average amount within the dataset. TIN-estimates ≥1.0, indicating twice as much aberrant exon skipping and inclusion as the average sample, and ≤ −1.0, indicating half as much as the average sample, are used as a thresholds for characterizing samples with TIN (TIN-samples). In comparisons across datasets, alternative splicing analyses by the FIRMA algorithm, and calculation of sample-wise TIN-estimates, were done across all the samples included in each comparison, as indicated.

### Statistical analyses

Statistical analyses were conducted in the R software environment (version 2.15.1). Permutations of TIN-estimates across the samples, and generation of random gene sets were done using the sample() function. Pearson correlations and two-sided Student *P*-values were calculated using the corAndPvalue() function in the WGCNA package [54]. Hierarchical clustering analyses with Euclidean distance metrics and complete linkage were done using J-Express 2011 (MolMine AS, Bergen, Norway). To corroborate the results from the clustering analyses, principle components analysis by singular value decomposition of the covariance matrix was performed using the prcomp() function in R. The component scores for the first two principal components are used to illustrate two-dimensional sample separation based on the input gene expression variables.

### Quality control of microarray data

The analyses presented in this study are potentially sensitive to the quality of the microarray data. Hence, exon-level quality control of all included samples was performed (Supplementary Methods in Additional file 1). All cancer (Additional file 1: Figure S5) and normal (Additional file 1: Figure S6) samples were preprocessed together and compared using the quality assessment metrics recommended by Affymetrix [55] and reported by the Affymetrix Expression Console™ software.

### Splicing factors and miscellaneous gene sets

We have created a comprehensive list of 280 splicing factor genes by combining search results from public annotation databases, as previously described [20]. Gene-level expression measures of these splicing factors were obtained from the CEL files of all samples included here. The CEL files were preprocessed across the datasets by the RMA approach [53], performing background correction, inter-chip quantile normalization, and gene-level summarization. For this, the Affymetrix Expression Console™ 1.1 software and Affymetrix HuEx-1_0-st-v2.r2 gene-core library files were used.

For comparison with the splicing factor genes, all the 1,454 Gene Ontology gene sets (the Gene Ontology Project [56]) collected in MSigDB [57] were also included. This collection of gene sets (ranging in size from 10 to 2,131 genes) comprises 8,282 unique genes, corresponding to 7,923 transcript clusters on the exon arrays (matched by gene symbols), thus representing 44% of the genes on the arrays. These gene sets are available from the ‘TIN’ analysis package.

Previously unpublished microarray data have been deposited to the NCBI’s Gene Expression Omnibus (GEO; accession numbers GSE42690 and GSE42954). R codes for alternative splicing analyses, statistical analyses, and plotting functions have been collected in a new Bioconductor package called ‘TIN’. This package is available for download from http://www.bioconductor.org/. A description of the package, with analysis protocols and R codes can also be found at this web site.

### Availability of supporting data

The data sets supporting the results of this article are available in the NCBI’s Gene Expression Omnibus repository, GSE42690 and GSE42954.

## Electronic supplementary material

Additional file 1: **Supplementary Methods, Table S1, Figures S1-S13.** (DOCX 1 MB)

## Abbreviations

FIRMA: Finding isoforms using robust multichip analysis
GEO: Gene expression omnibus
mRNA: Messenger RNA
MSI: Microsatellite instability
MSigDB: The molecular signatures database
MSS: Microsatellite stable
NA: Not available
T: Tumor
TIN: Transcriptome instability.
