# Supplementary material for: Transcriptome instability as a molecular pan-cancer characteristic of carcinomas
Source: BMC Genomics. 2014 Aug 10;15(1):672. doi: 10.1186/1471-2164-15-672 (PMC4137096; doi:10.1186/1471-2164-15-672)
Supplement: Supplementary file 1 — Additional file 1: Supplementary Methods, Table S1, Figures S1-S13. (DOCX 1 MB) [file 12864_2014_6357_MOESM1_ESM.docx]

## Additional file 1:

This file contains Supplementary Methods, Table S1, and Figures S1-S13

## Supplementary Methods

### Quality control of microarray data

To facilitate quality control comparisons across the datasets, all cancer samples (n = 555) and normal samples (n = 93) were preprocessed together. Raw data was preprocessed and summarized on the exon (probe set) level by the robust multi-array average approach, using the Affymetrix Expression Console^TM^ 1.1 software. The quality assessment metrics recommended by Affymetrix were tested (http://media.affymetrix.com/support/technical/whitepapers/exon_gene_arrays_qa_whitepaper.pdf). Mean absolute relative log expression (RLE; expression signal for a probe set relative to the median of that probe set across all samples; log2 scale) was calculated for all probe sets, as well as for positive controls (probe sets targeting exons of housekeeping genes) and negative controls (probe sets targeting introns of housekeeping genes) per sample. The present call (percentage of probes detected above the background level) and area under the curve (AUC) for receiver operating characteristic plots, comparing expression of positive and negative controls, were also calculated. Lastly, the relative expressions of two sets of spike-in controls were compared. The bacterial spike hybridization controls are added to the hybridization cocktail with increasing concentrations in the following order: BioB, BioC, BioD, Cre. The poly-A spike amplification controls are added to the RNA sample with increasing concentrations in this order; Lys, Phe, Thr, Dap. The expression signals for the probes within the two control sets should increase correspondingly.

In general, there were good and corresponding results for the quality assessment metrics between the datasets. Among the cancers, the cervical cancer dataset showed most deviating results (Figure S5). In particular, the AUC values for the positive *vs*. negative controls were low in this dataset, ranging from 0.62 to 0.75 (should according to Affymetrix’ guidelines typically range from 0.8 to 0.9), and the mean absolute RLE for all probe sets was higher than for the rest of the samples. All the normal sample datasets were of good quality, although the stomach dataset showed a small increase in mean absolute RLE-values for all probe sets, as well as positive and negative controls, compared with the other datasets, and also a somewhat lower present call (Figure S6).

### Table S1. Results from the alternative splicing (FIRMA) analyses

| Dataset | No. of samples | Median FIRMA score (log2) | FIRMA score threshold value (log2) | | TIN-estimates (log2) | | | No. of TIN-samples | |
| --- | --- | --- | --- | --- | --- | --- | --- | --- | --- |
|  |  |  | 1st percentile | 99th percentile | Min | Max | Range |  |  |
| Breast cancer | 84 | 0.02 | -2.4 | 2.1 | -1.6 | 2.8 | 4.4 | | 23 (27%) |
| Cervical cancer | 28 | 0.01 | -2.1 | 2.0 | -1.3 | 1.5 | 2.8 | | 5 (18%) |
| Colorectal cancer I | 26 | 0.01 | -2.2 | 1.9 | -1.7 | 1.7 | 3.4 | | 8 (31%) |
| Colorectal cancer II | 101 | 0.02 | -2.4 | 2.1 | -1.6 | 1.7 | 3.3 | | 25 (25%) |
| Gastric cancer | 25 | 0.007 | -2.1 | 1.9 | -1.5 | 1.2 | 2.7 | | 3 (12%) |
| Lung cancer I | 20 | 0.02 | -2.3 | 1.9 | -1.0 | 0.9 | 1.9 | | 0 |
| Lung cancer II | 43 | 0.02 | -2.4 | 2.0 | -1.7 | 1.7 | 3.4 | | 17 (40%) |
| Neuro-blastoma | 47 | 0.02 | -2.3 | 2.0 | -1.8 | 1.8 | 3.6 | | 9 (19%) |
| Prostate cancer I | 131 | 0.008 | -2.1 | 2.0 | -3.1 | 2.4 | 5.5 | | 63 (48%) |
| Prostate cancer II | 50 | 0.02 | -2.4 | 1.9 | -1.3 | 1.9 | 3.2 | | 5 (10%) |
| Normal colonic mucosa | 21 | 0.01 | -2.2 | 1.9 | -1.1 | 1.6 | 2.7 | | 7 (33%) |
| Normal lung | 20 | 0.02 | -2.3 | 1.9 | -1.1 | 0.9 | 2 | | 1 (5%) |
| Normal prostate | 29 | 0.01 | -2.2 | 2.0 | -2.2 | 1.8 | 4 | | 10 (34%) |
| Normal stomach | 23 | 0.004 | -2.0 | 1.8 | -1.9 | 2.2 | 4.1 | | 6 (26%) |

FIRMA, Finding Isoforms using Robust Multichip Analysis (Purdom *et* *al*., 2008, *Bioinformatics*); TIN-estimates, amounts of aberrant exon inclusion and skipping per sample (on a log2-scale), relative to the average amount within the dataset; TIN-samples, samples with TIN-estimates > ±1.0


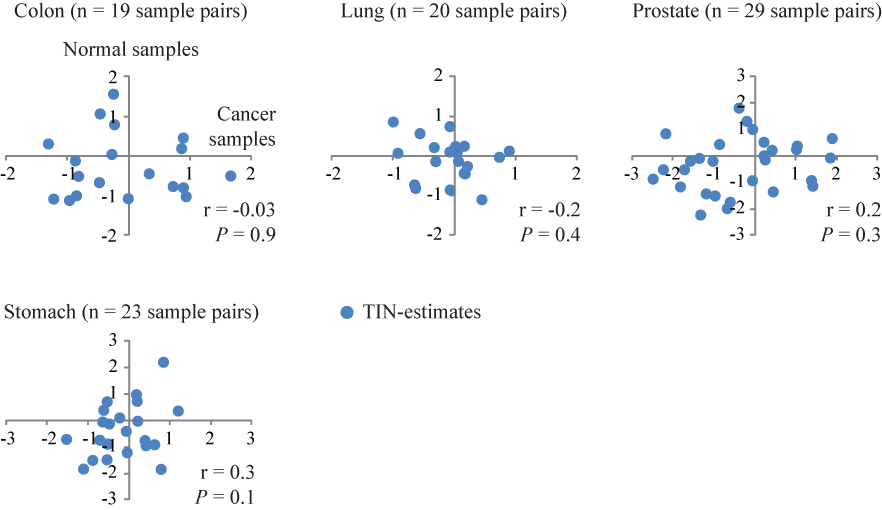


### Figure S1. Correspondence in TIN-estimates between paired cancer and normal samples

There was no correspondence in TIN-estimates between paired normal and cancer samples in any of the four tissue types analyzed.


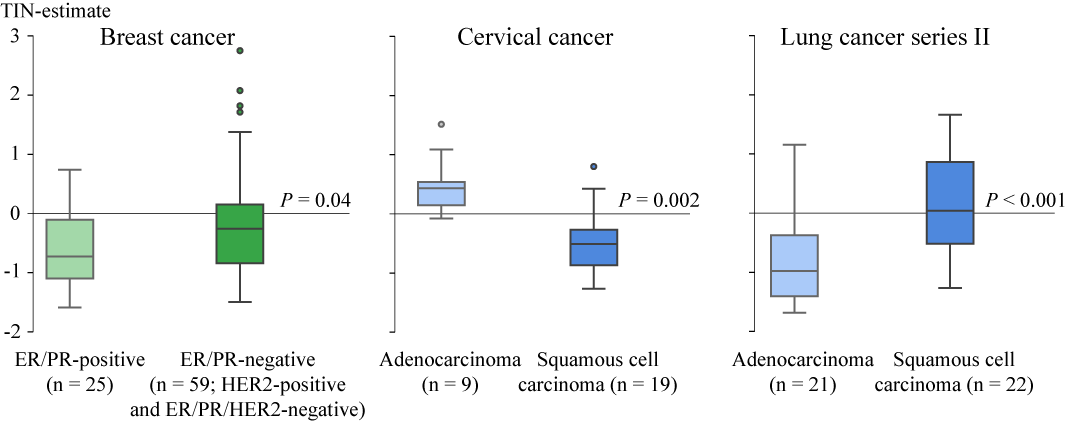


### Figure S2. Differences in TIN-estimates between cancer subgroups

In the breast cancer dataset, ER/PR-positive samples had significantly lower TIN estimates than ER/PR-negative samples. In both the cervical cancer dataset and lung cancer series II, there were significant differences in TIN-estimates between adenocarcinomas and squamous cell carcinomas, but in opposite directions.


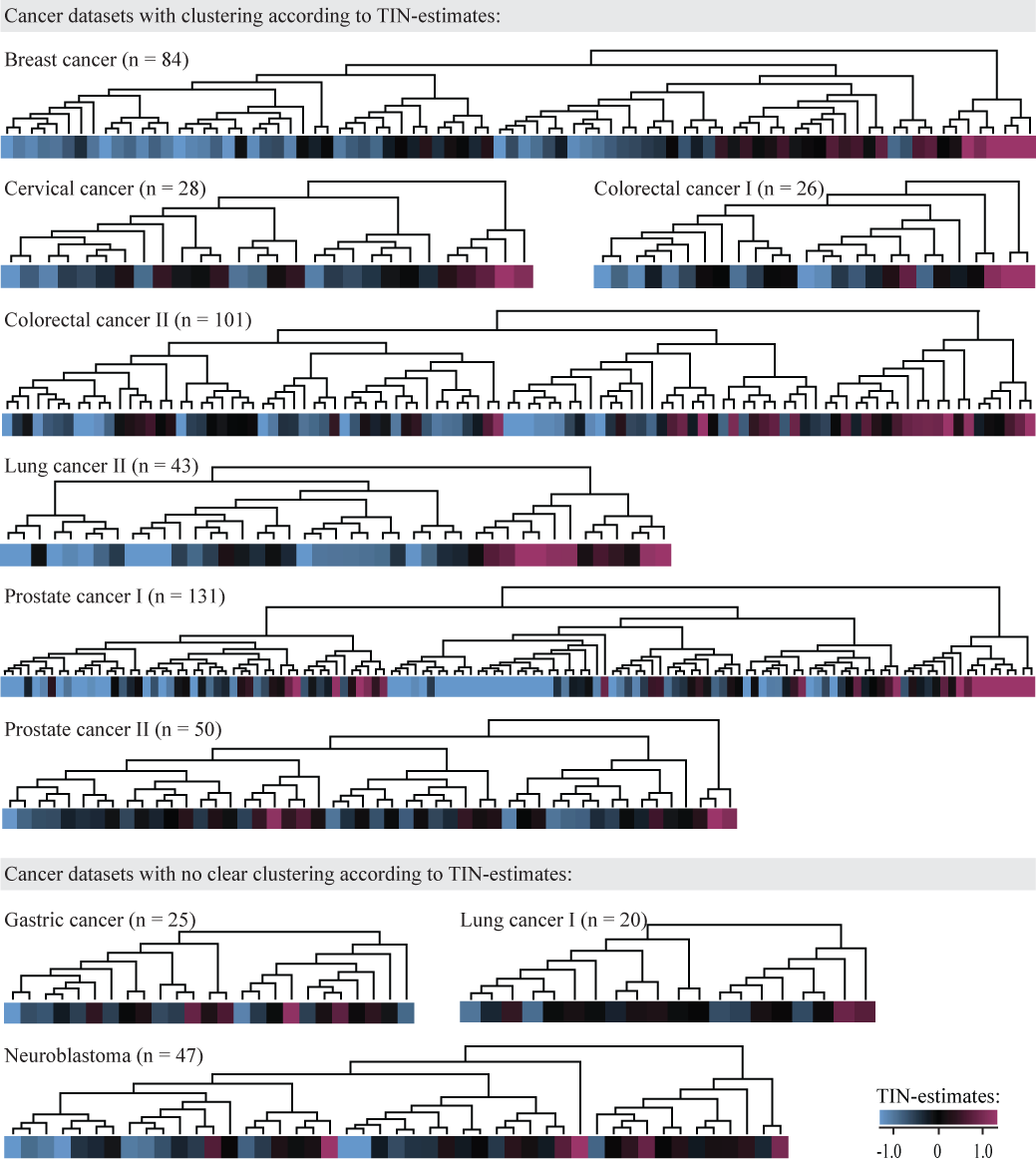


### Figure S3. Hierarchical clustering analyses of cancer datasets by the expression level of splicing factors

Unsupervised hierarchical clustering analyses by the expression levels of splicing factors (n = 280) of all the samples from breast cancer, cervical cancer, colorectal cancer series I and II, lung cancer series II, and prostate cancer series I and II separated the samples into clusters with predominantly high (red) and low (blue) TIN-estimates (upper panel). In contrast, there was no clear separation of samples in the datasets from gastric cancer, lung cancer series I, and neuroblastoma (bottom panel). The hierarchical clustering analyses were performed by Euclidean distance metrics and complete linkage.


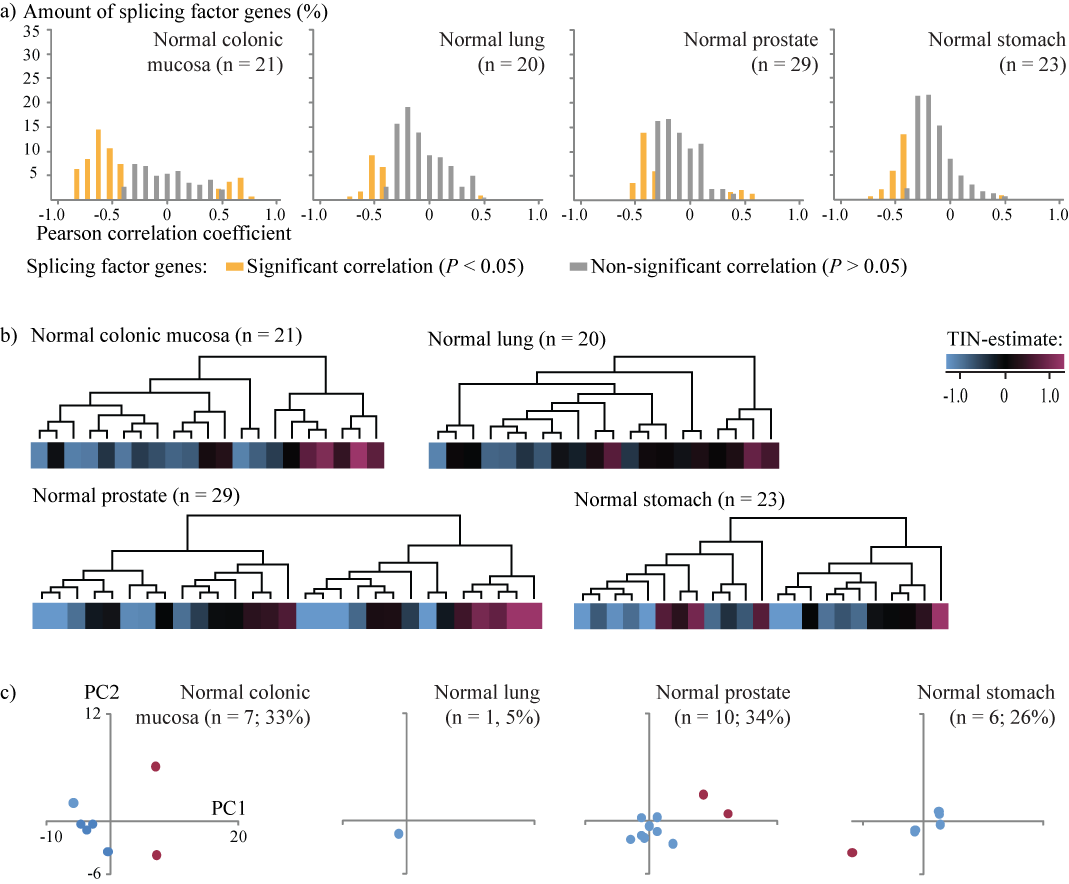


### Figure S4. Associations between TIN-estimates and expression levels of splicing factors in the normal tissue types

a) In the normal tissue types, 18% (lung) to 56% (colonic mucosa) of the splicing factor genes (n = 280) had expression levels that were significantly (yellow bars) correlated with the sample-wise TIN-estimates. (b) In concordance with colorectal cancer series II and prostate cancer series I, paired normal samples from colonic mucosa and the prostate were separated into clusters with predominantly high (red) and low (blue) TIN-estimates upon hierarchical clustering analyses by the expression levels of splicing factor genes. Also corresponding with the gastric and lung cancer samples, paired normal samples from the lung and stomach showed poorer separation according to TIN-estimates. The hierarchical clustering analyses were performed by Euclidean distance metrics and complete linkage. (c) Corresponding with the clustering analyses, principal components analysis based on the expression levels of the splicing factor genes, separated samples from the normal colonic mucosa and normal prostate according to TIN-estmates (only samples with TIN-estimates ≥±1.0 were included, the number and percentages of samples are indicated for each dataset). In the normal lung and stomach datasets, there were few samples with TIN-estimates ≥ ±1.0.

PC, principal component


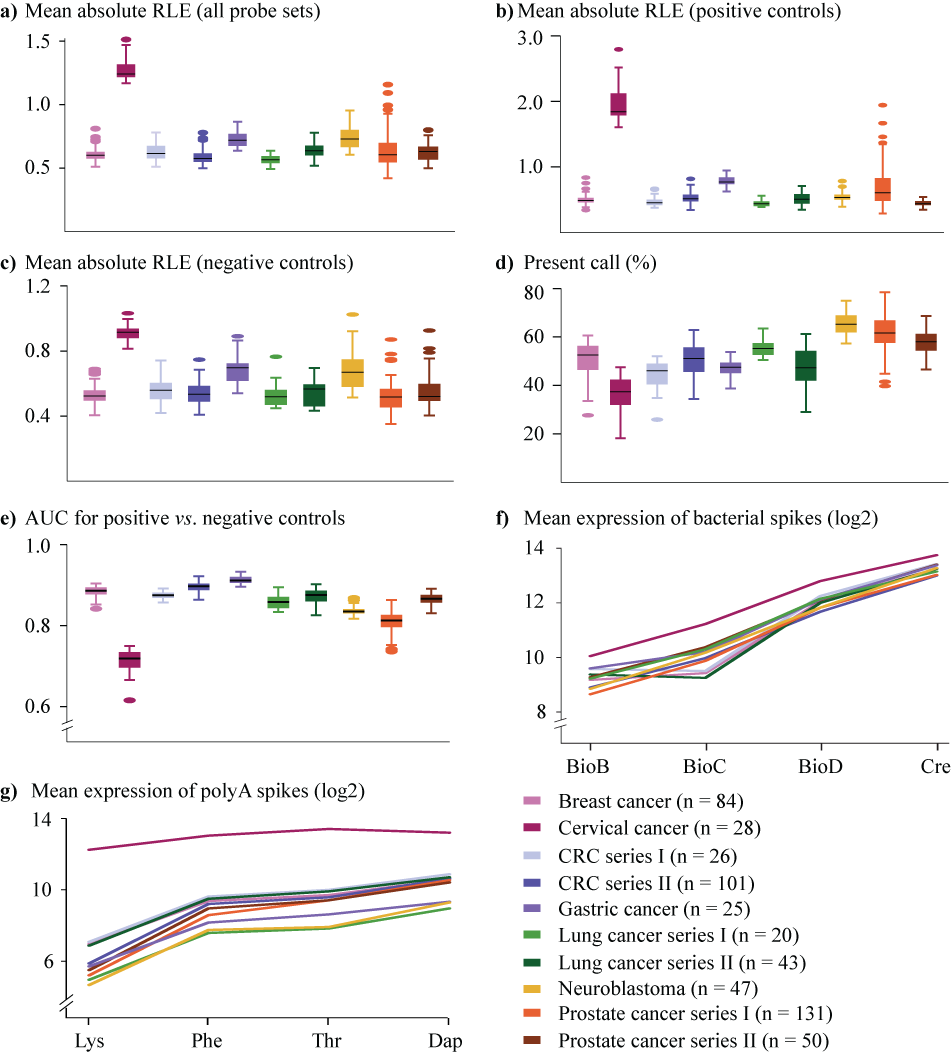


### Figure S5. Quality control of microarray data for all the cancer datasets

Quality control of expression data preprocessed across all samples in the nine cancer datasets (total n = 555), and summarized at the exon level, showed good and corresponding control metrics for all datasets, except for the cervical cancer dataset. Mean absolute RLE values (log2 scale) for (a) all probe sets, (b) positive controls (exons of housekeeping genes) and (c) negative controls (introns of housekeeping genes) were higher in the cervical cancer dataset. Both (d) the present call and (e) the AUC were lower in the cervical cancer dataset compared with the other datasets. Mean expression levels of both (f) bacterial spike controls and (g) polyA spike controls (hybridization and amplification controls added to the hybridization cocktail and RNA samples, respectively) increased with increasing concentrations in all datasets. AUC, area under the curve for receiver operating characteristic plots; RLE, relative log expression (expression signal for a probe set relative to the median of the particular probe set across all samples).


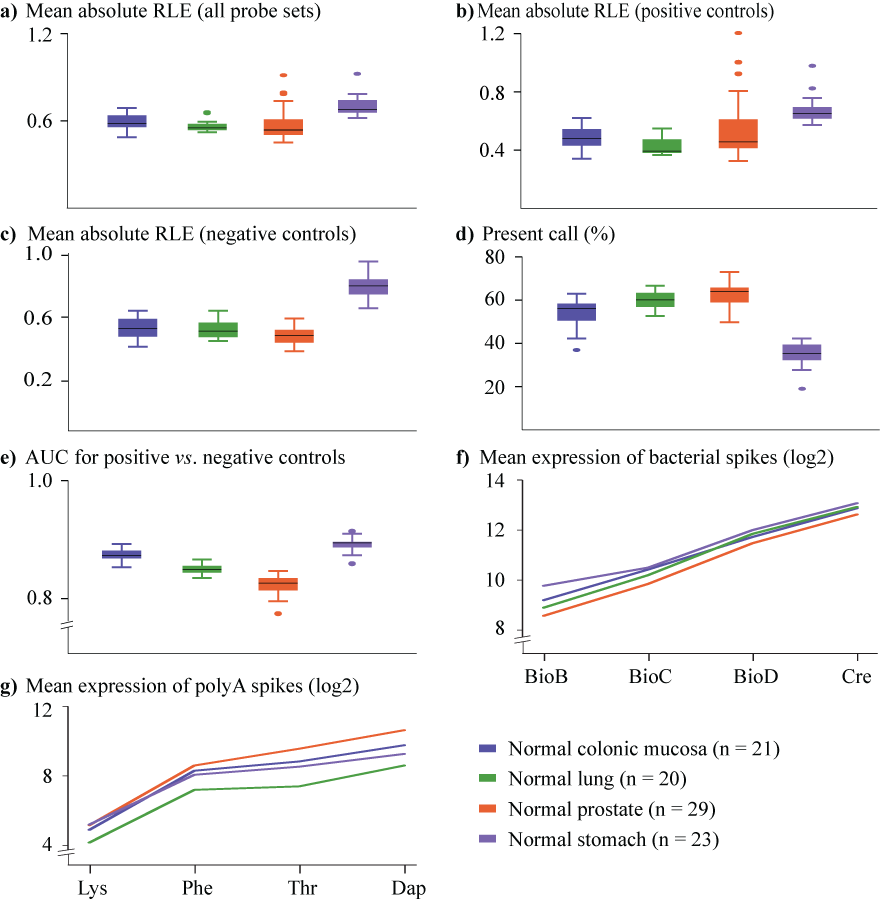


### Figure S6. Quality control of microarray data for all the normal tissues

Quality control of expression data preprocessed across all normal samples (n = 93) and summarized at the exon level, showed good and corresponding control metrics for all datasets. Mean absolute RLE values (log2 scale) for (a) all probe sets, (b) positive controls (exons of housekeeping genes) and (c) negative controls (introns of housekeeping genes) were somewhat higher in the stomach dataset compared with the other datasets, and (d) the present call was lower. (e) The AUC values were >0.8 in all datasets. Mean expression levels of both (f) bacterial spike controls and (g) polyA spike controls (hybridization and amplification controls added to the hybridization cocktail and RNA samples, respectively) increased with increasing concentrations in all datasets. AUC, area under the curve for receiver operating characteristic plots; RLE, relative log expression (expression signal for a probe set relative to the median of the particular probe set across all samples).


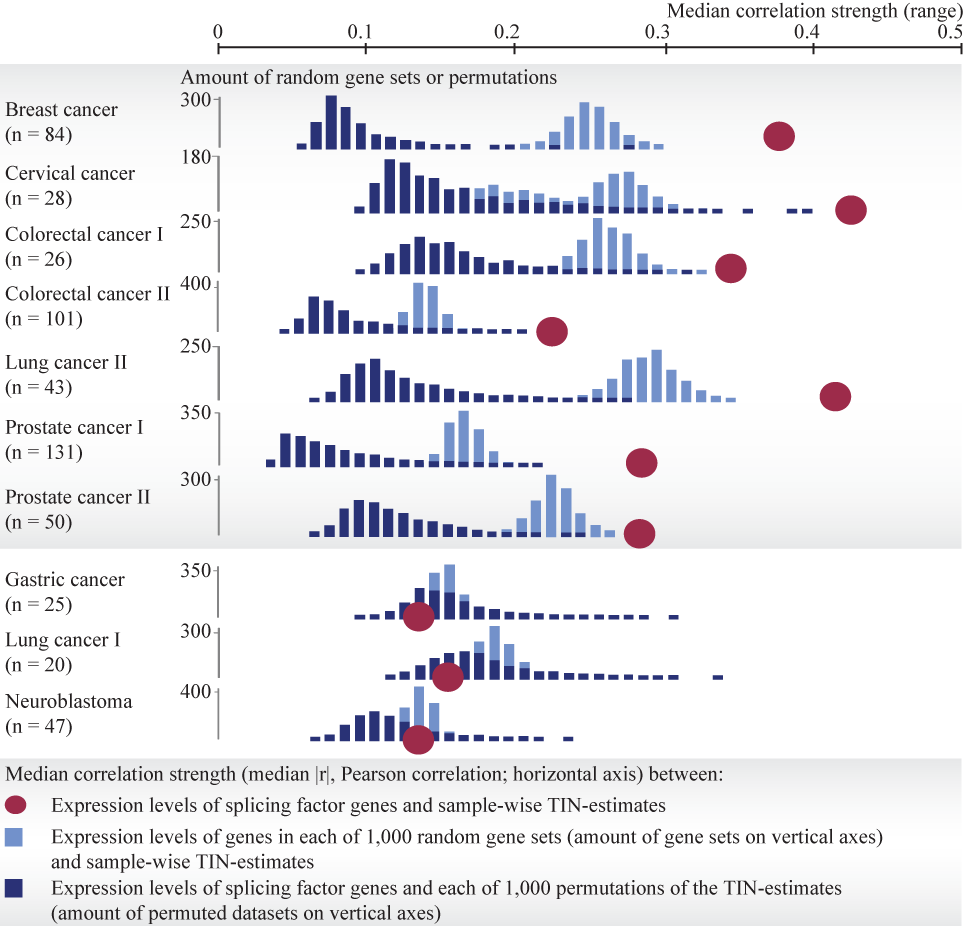


### Figure S7. Correlation strengths between expression levels of splicing factors and TIN-estimates in the cancer datasets

In seven of the cancer datasets (with grey background), the median correlation strengths (absolute values for the Pearson correlation coefficients; red dots) between the expression levels of 280 splicing factor genes and the sample-wise TIN-estimates was higher than expected by chance, compared with genes in each of 1,000 random sets of 280 genes (bar graphs in light blue) and with 1,000 permutations of TIN-estimates (bar graphs in dark blue). In the other three cancer datasets (no background), the median correlation strengths were low, and not higher than expected by chance.


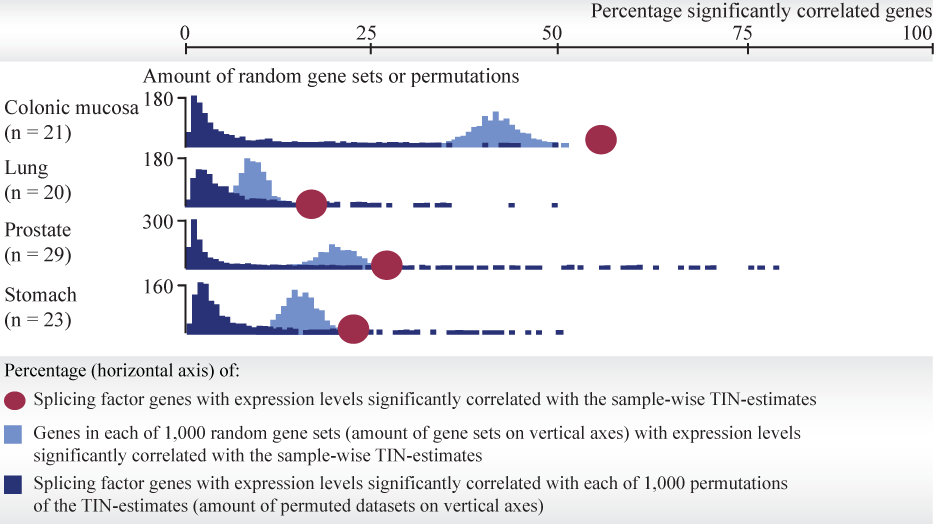


### Figure S8. Correlation between TIN-estimates and splicing factor expression compared with random distributions in the normal tissues

In the normal tissues, the percentages of the 280 splicing factor genes (red dots; horizontal axis) that had expression levels significantly correlated (*P* < 0.05; Pearson correlation) with the TIN-estimates, were higher than can be expected by chance (compared with genes in each of 1,000 random sets of 280 genes (bar graphs in light blue) and with 1,000 permutations of the TIN-estimates (bar graphs in dark blue)).


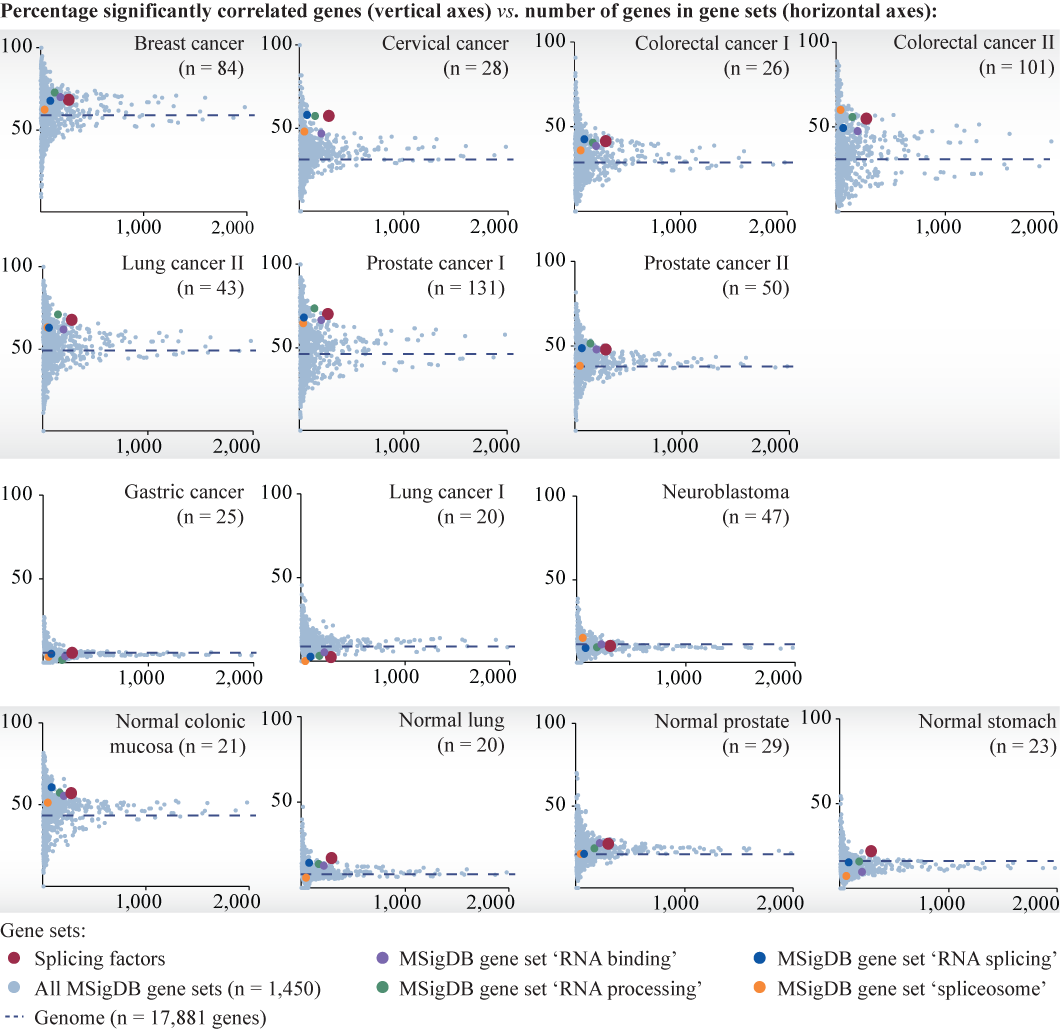


### Figure S9. Percentage significantly correlated genes in the splicing factor gene set and miscellaneous gene sets in all cancer datasets and normal tissues

The percentage of genes (vertical axes) in the splicing factor gene set (red; n = 280 genes) that was significantly correlated with the TIN-estimates, was high in seven cancer datasets (grey background, upper panel), compared with Gene Ontology gene sets in MSigDB (pale blue) and the full genome (dashed line), seen in relation to the amounts of genes in each gene set (horizontal axes). Splicing-related gene sets in MSigDB (colored as indicated) also had high percentages of significantly correlated genes in these cancer datasets. Contrarily, in the other three cancer datasets (no background, middle panel), none of the splicing-related gene sets had high percentages of significantly correlated genes compared with the miscellaneous gene sets. In the normal tissues (grey background, lower panel), the splicing-related gene sets generally had high percentages of significantly correlated genes compared with miscellaneous gene sets. MSigDB, Molecular Signatures Database.


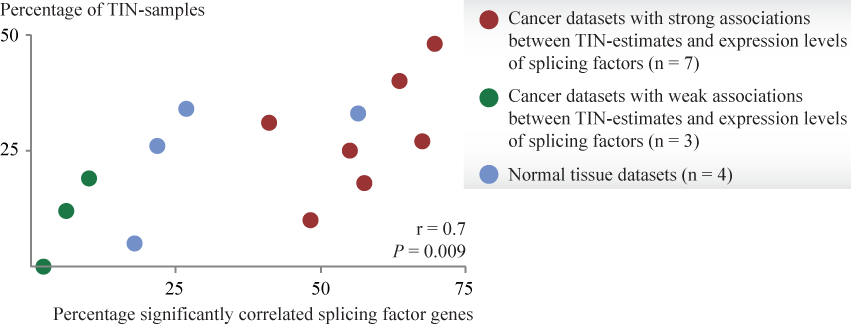


### Figure S10. Correspondence between the extent of splicing variation and correlation with expression levels of splicing factors

There was a proportional relationship between the percentages of TIN-samples in the datasets and the percentage of splicing factors with expression levels significantly correlated with the TIN-estimates. In the cancer datasets shown to have strong associations between the TIN-estimates and the expression levels of splicing factors (red), there were generally a high percentage of TIN-samples, while the cancer datasets with weak associations had few TIN-samples. The normal tissue datasets (blue) had intermediate percentages of TIN-samples. The normal colonic mucosa dataset clustered among the TIN-cancer datasets in this regard, corresponding with the strong relationship between TIN-estimates and expression levels of splicing factors in this dataset compared with the other normal tissue datasets.

TIN-samples, samples with TIN-estimates > ±1.0


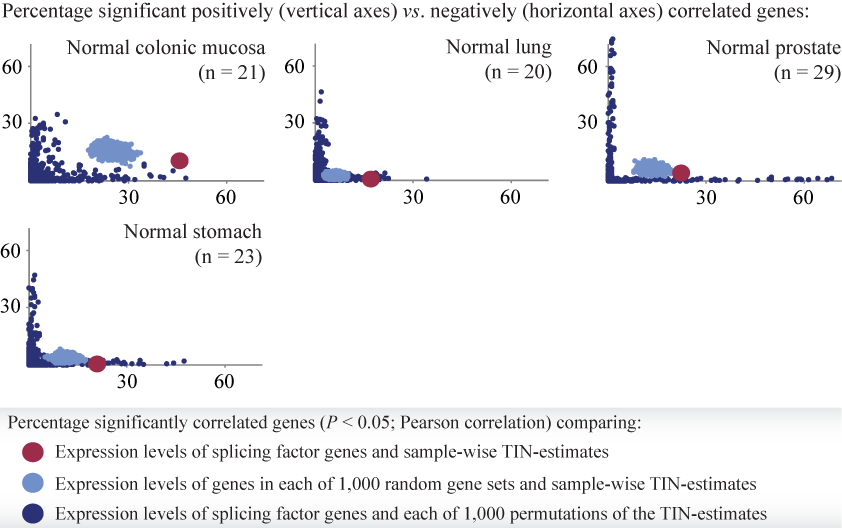


### Figure S11. Negative correlation between TIN-estimates and splicing factor expression in the normal tissues

There was an inverse relationship between the TIN-estimates and expression levels of splicing factor genes (n = 280) in the normal tissue types, with a higher percentage of significant negatively (horizontal axes) than positively (vertical axes) correlated genes (red). This shift was higher than expected by chance, as compared with genes in each of 1,000 random sets of 280 genes (light blue), and with each of 1,000 permutations of the TIN-estimates (dark blue).


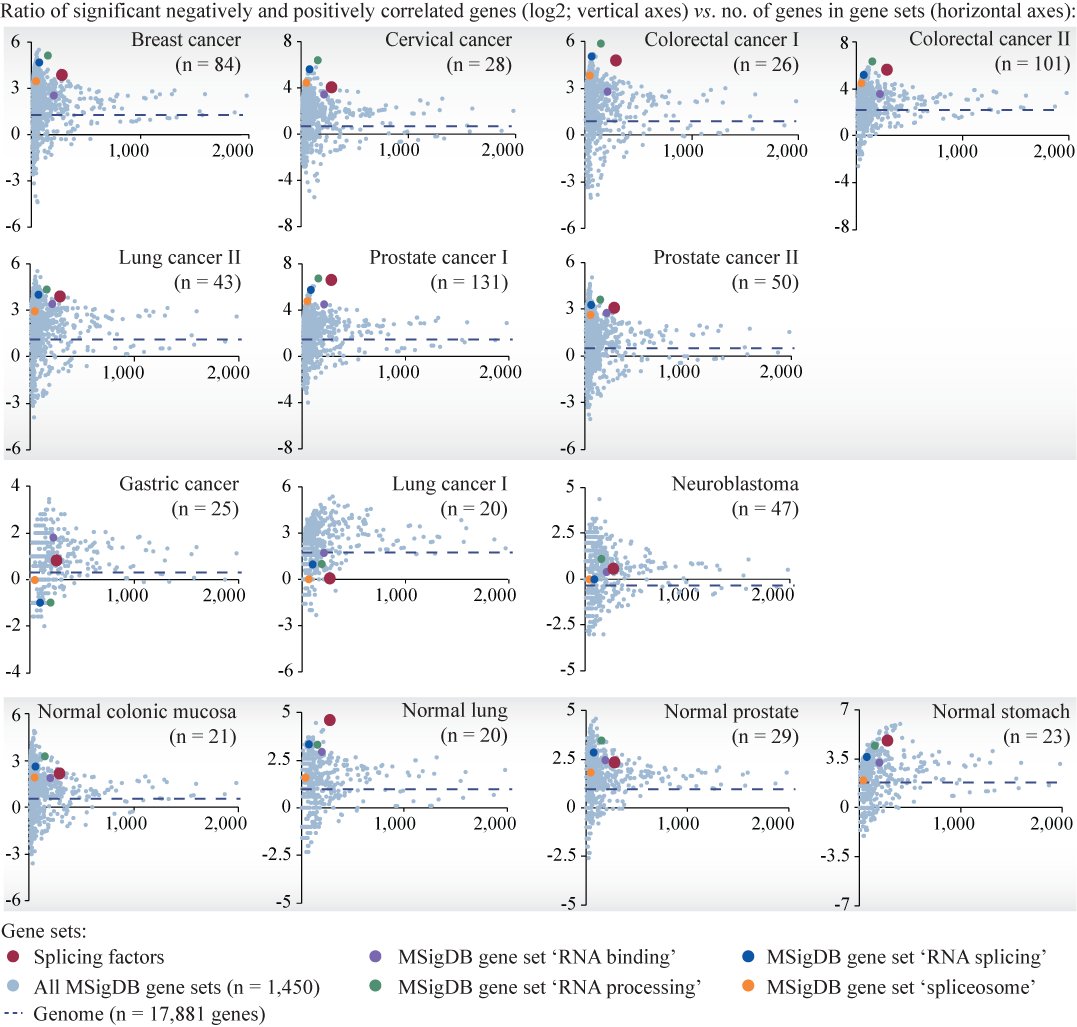


### Figure S12. Ratio of significant negatively and positively correlated genes in the splicing factor gene set and miscellaneous gene sets in all cancer datasets and normal tissues

The ratio of significant negatively *vs*. positively correlated genes (log2; vertical axes) in the splicing factor gene set (red; n = 280 genes) was high in seven cancer datasets (grey background, upper panel), compared with Gene Ontology gene sets in MSigDB (pale blue) and the full genome (dashed line), seen in relation to the amounts of genes in each gene set (horizontal axes). Splicing-related gene sets in MSigDB (colored as indicated) also had high amounts of significant negatively *vs*. positively correlated genes in these cancer datasets. Contrarily, in the other three cancer datasets (no background, middle panel), none of the splicing-related gene sets had strong shifts towards negative correlation compared with the miscellaneous gene sets. In the normal tissues (grey background, lower panel), the splicing-related gene sets generally had high ratios of significant negatively *vs*. positively correlated genes.

MSigDB, Molecular Signatures Database.


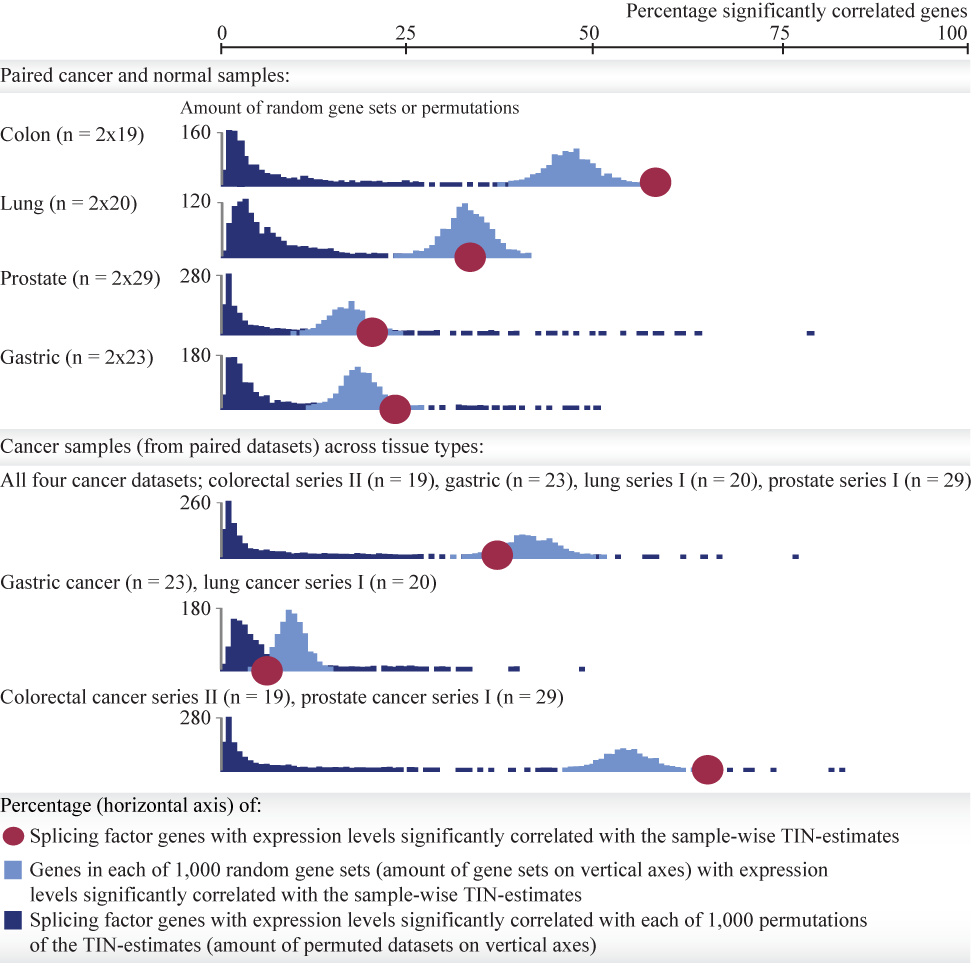


### Figure S13. Associations between TIN-estimates and expression levels of splicing factors in analyses across cancer and normal tissue types

In analyses of paired cancer and normal samples (upper panel), the percentages of splicing factor genes (totally 280 genes, red dots, horizontal axis) with expression levels that were significantly correlated (*P* < 0.05; Pearson correlation) with TIN-estimates, were not higher than expected by chance (except for in the colon; compared with genes in each of 1,000 random sets of 280 genes (bar graphs in light blue) and with 1,000 permutations of the TIN-estimates (bar graphs in dark blue)). This is compliant with cancer specificity in splicing patterns. The same was seen when analyzing across all the cancer samples from the same datasets (lower panel), as well as across the two cancer datasets that each individually had weak associations between TIN-estimates and splicing factor expression (gastric and lung cancer). Again, the failure to detect a common splicing pattern is compliant with tissue specificity. However, when analyzing across the two cancer datasets that individually showed strong associations between TIN-estimates and splicing factor expression (colorectal and prostate cancer), the percentage of splicing factor genes with expression levels that were significantly correlated was stronger than expected by chance, indicating that these cancer types fail to adhere to splicing patterns consistent with tissue specificity.
